# Diversity and Dynamics of Yeasts During Vidal Blanc Icewine Fermentation: A Strategy of the Combination of Culture-Dependent and High-Throughput Sequencing Approaches

**DOI:** 10.3389/fmicb.2019.01588

**Published:** 2019-07-09

**Authors:** Jing Li, Wen-Zhong Hu, Yong-Ping Xu

**Affiliations:** ^1^School of Life Science and Biotechnology, Dalian University of Technology, Dalian, China; ^2^Institute of Food Science and Engineering, Jinzhou Medical University, Jinzhou, China; ^3^College of Life Science, Dalian Minzu University, Dalian, China

**Keywords:** icewine, yeast, dynamics, spontaneous and inoculated fermentations, culture-dependent method, high-throughput sequencing

## Abstract

In this study, attention has been focused on the ecology of yeasts during the spontaneous and inoculated fermentation processes of Vidal blanc icewine in northeast China, which is very important for screening autochthonous yeast strains, understanding the roles of these strains, and managing fermentation. The strategies were to conduct spontaneous and inoculated laboratory-scale fermentation processes simultaneously and to analyze the samples taken at different fermentation stages by culture-dependent and -independent methods. Three hundred and thirty-eight yeast strains were isolated and twelve genera were identified by sequencing. During the spontaneous fermentation process, non-*Saccharomyces* yeasts were predominant in the initial and middle stages, whereas *Saccharomyces* dominated in the later stages; *Candida* was preponderant in the whole process, and its abundance in the final stages was only lower than *Saccharomyces.* The inoculated fermentation was characterized by a predominance of *Saccharomyces* throughout the fermentation process; non-*Saccharomyces* yeasts were observed in the early stage. The internal transcribed spacer (ITS) 2 region gene was firstly used to analyze the yeast diversity in the samples during the icewine fermentation processes by high-throughput sequencing (HTS), and a more complex mycobiota was revealed. Moreover, the dynamics of other major fungi (mainly *Davidiella* and *Alternaria*) during icewine fermentation processes were also revealed, which have never been reported in icewine before.

## Introduction

Icewine originated in Germany more than 200 years ago. It is a typical dessert wine made from grapes that have naturally frozen on the vine under cold weather conditions (below −8°C) (OIV, 2008). Due to the specific environmental conditions required for planting and harvesting ice grapes, icewine is manufactured only in a few countries where winter conditions are cold enough. Canada, Germany, and Austria are the world’s major producers of icewine (Tian et al., 2009). In recent years, the icewine industry in China has developed rapidly, and China has become an icewine production country that cannot be ignored (Alessandria et al., 2013; Ma et al., 2017; Huang et al., 2018). Vidal blanc grapes have a high level of winter hardiness because of thick-skinned berries (Bowen and Reynolds, 2015); it is the main cultivar for icewine production in the northeast China. Vidal blanc icewine products are considered to be of high quality because of their appealing aroma and attractive flavor (Nurgel et al., 2004; Crandles et al., 2015).

Alcoholic fermentation of grape juice is a complex biochemical process carried out by a dynamically changing microbiota, in which yeast strains play an important role. Icewine yeasts need to face many challenges during the fermentation process, e.g., high sugar concentrations, high acid concentrations and low fermentation temperature (Zhang et al., 2018). During the process of conversion of ice grape juice into icewine, the composition changes dramatically under the action of *Saccharomyces* and non-*Saccharomyces* yeasts. Several studies have shown that non-*Saccharomyces* yeast contributes positively to the aroma and flavor of wine (Fleet, 2003; Ciani et al., 2010; Lencioni et al., 2016; Englezos et al., 2018) and icewine (Zhang et al., 2018). A few non-*Saccharomyces* yeast genera were reported to adequately produce hydrolytic enzymes, which can facilitate the release of aromatic precursors (Pérez et al., 2011; Maturano et al., 2012).

Due to the easy operation and stability of product quality, commercial yeast strains are currently used to inoculate icewine fermentation in many wineries. Another strategy is to favor the screening and utilization of autochthonous yeast strains, because these yeasts can adapt well to the micro-conditions of the wine region and generate territorial characteristics in icewine products (Zhang et al., 2018). Therefore, the knowledge of the yeast diversity and dynamics during inoculated and spontaneous fermentations is very important for controlling fermentation, screening autochthonous yeast strains and understanding the role of these yeast strains in the process. However, most studies have focused on the aroma compounds and flavor characteristics of icewine (Bowen and Reynolds, 2015; Ma et al., 2017; Huang et al., 2018), and the knowledge of the microbiota involved in the fermentation of icewine is very limited.

Thus far, yeast diversity and dynamic changes during the fermentation process have been researched by culture-dependent and -independent methods. The culture-dependent approach using the Wallerstein laboratory (WL) nutrient agar medium has been employed to monitor and isolate the yeasts during grape juice fermentation based on the colors and morphologies of the colonies (Cavazza et al., 1992; Urso et al., 2008; Alessandria et al., 2013). For further identification of yeast species, the 5.8S-ITS region gene or the D1/D2 domain of the 26S rDNA gene has been sequenced (Brysch-Herzberg and Seidel, 2015). In recent years, PCR-DGGE (Alessandria et al., 2013; Bučková et al., 2018) and HTS (Andorrà et al., 2010; De Filippis et al., 2017; Mendoza et al., 2017) as culture-independent methods have been used for detecting yeast population diversity. In general, in terms of supporting the results of the culture-dependent methods, culture-independent methods have detected higher microbial diversity (Van Hijum et al., 2013). HTS has been shown to have better potential for analyzing the microbial diversity of fermented foods as compared to PCR-DGGE (Połka et al., 2015; Portillo and Mas, 2016). However, in this decade, studies on the microbiota involved in the fermentation of icewine were only carried out by using PCR-DGGE as a culture-independent method by Alessandria et al. (2013) and Bučková et al. (2018).

The main objective of this study was to comprehensively analyze yeast population diversity and dynamics during the spontaneous and inoculated fermentation processes of Vidal blanc icewine of northeast China by culture-dependent (WL nutrient agar) and culture-independent (HTS) approaches.

## Materials and Methods

### Laboratory-Scale Fermentation Processes and Sampling

The ice grape berries of Vidal blanc used in this study were harvested manually from the vineyard of “Sun Valley” winery (NE China, 41°45′24.97″N 123°62′20.58″E) in December 2016. Healthy ice grapes were randomly collected, and the peduncles and leaves were fully removed. The frozen grapes were then pressed to obtain ice grape juice for fermentative production of icewine. The ice grape juice was immediately placed in sterile buckets (at ≤ 0°C) and transported to the laboratory. The ice grape juice was named SZ and found to have pH 3.89 and sugar content of approximately 27.3° Brix. Laboratory-scale spontaneous and inoculated fermentation processes were carried out simultaneously. Spontaneous fermentation processes were carried out with 600 mL of the ice grape juice (containing 50 mg/L SO_2_) in 1000-mL sterile flasks in triplicate at 18°C under static conditions. Inoculated fermentations were performed in a similar way, although in addition commercial active dried yeast (ST; LAFFORT, Bordeaux, France; initial inoculum of approximately 10^6^ cells/mL final concentration) was added to the ice grape juice. Samples consisted of longitudinal icewine fermentation samples, and sampling was carried out at 0, 1, 2, 4, 7, 14, 21, and 30 days during the spontaneous and inoculated fermentation processes. The samples of the spontaneous fermentation were named SSFN-[1, 2, 3] (N represented sequential sampling points, N = A, B…G), while samples from the inoculated fermentation were designated SJFN-[1, 2, 3] (N = A, B…G). Microbial cultivation and DNA extraction procedures were performed for each sample.

At the end of fermentation, the means of pH, ethanol, and sugar content in the icewines produced by the spontaneous and inoculated fermentation processes were determined. The pH was determined using a pH-meter (pHS-3C; WEIYE, Shanghai, China), and the sugar content was determined using OIV methods (OIV, 2008). The ethanol was quantified using gas chromatograph with a flame ionization detector (GC 9790 plus; Fuli, Zhejiang, China), and the system was equipped with a KB-5 capillary column (30 m × 320 μm × 0.25 μm; Kromat Corporation, Bordentown, NJ, United States); the column temperature was 50°C, the injection port was 250°C, and the detector temperature was 250°C; ethanol (GC quality standard reagents; Aladdin, Shanghai, China) as a standard was used; each sample of 0.2 μL was injected into the injection port, the carrier gas was nitrogen with the flow rate of 1 mL/min, and the injection split ratio was 50:1.

### Yeast Isolation and Cultivation

Each sample was serially diluted in a sterile physiological solution (with ratios of 1:10 to 1:10^6^) and spread-plated on WL Nutrient agar (Haibo, Qingdao, China). All plates were incubated at 28°C for 5 days. The colonies on each plate were counted, and mean values were calculated. At least 20 colonies with different colors and morphologies were selected and isolated from each plate as representatives at each sampling point of the fermentations. All pure isolates were cultured in YPD medium (yeast extract 10 g/L, peptone 20 g/L, dextrose 20 g/L; Haibo, Qingdao, China) at 28°C for 2 days and stored at −80°C after addition of glycerol (30% v/v).

### Molecular Identification of the Isolates

The stored isolate was activated in the YPD medium at 28°C for 2 days, and genomic DNA was extracted by means of the yeast genomic DNA extraction kit (DP307-02; TIANGEN, Beijing, China). DNA was quantified on an ND-1000 spectrophotometer (NanoDrop, Wilmington, DE, United States), and concentration was standardized to 100 ng/μL. The ITS region (ITS1-5.8S-ITS2) was amplified with primers ITS1 (5′-TCC GTA GGT GAA CCT GCG G-3′) and ITS4 (5′-TCC TCC GCT TAT TGA TAT GC-3′) (Brysch-Herzberg and Seidel, 2015). PCR was carried out in a final volume of 25 μL, and the composition of the PCR mixture was as follows: 2.5 U Taq DNA polymerase, 2.5 μL 10 × Taq buffer, 2 μL 25 mM MgCl_2_, 0.5 μL of each dNTP (10 mM), 1 μL of 10 nM forward primer and 1 μL of 10 nM reverse primer, and 1 μL of the template DNA. Thermal cycling was performed as follows: initial denaturation at 95°C for 5 min; 35 cycles (95°C for 30 s, 55°C for 30 s, 72°C for 1 min); and a final elongation at 72°C for 7 min. When necessary, the 26S rDNA D1/D2 domain sequences of the isolates were additionally sequenced and analyzed (Fell et al., 2000). This region was amplified with the universal primer pairs NL1 (5′-GCATATCAATAAGCGGAGGAAAAG-3′) and NL4 (5′-GGTCCGTGTTTCAAGACGG-3′) (Brysch-Herzberg and Seidel, 2015; Graça et al., 2015); in addition, the amplification reaction and the PCR conditions were identical to those described for the ITS region (Santo et al., 2012). All PCR products of extracted DNA samples were sent to Sangon Biotech Co., Ltd (Shanghai, China) for sequencing. For identification of the isolates, these sequences were edited in the MEGA software (version 6.06) (Tamura et al., 2013) and compared with the present in the NCBI database (National Centre for Biotechnology Information^1^) using BLAST (Basic Local Alignment Search Tool) search to determine the closest known relatives.

### Illumina HTS

The total DNA of each sample was directly extracted with the E.Z.N.A. ^®^Soil DNA Kit (D5625, Omega, Inc., United States). DNA extracts were sent to LC-Bio Technology Co., Ltd. (Hangzhou, Zhejiang Province, China) for PCR amplification and HTS. The internal transcribed spacer2 (ITS2) region of high-quality DNAs of the samples was selected (Pinto et al., 2015) and amplified with slightly modified versions of primers fITS7 (5′-GTGARTCATCGAATCTTTG-3′) and ITS4 (5′-TCCTCCGCTTATTGATATGC-3′). The 5′ ends of the primers were tagged with specific barcodes and sequencing universal primers. PCR was carried out in a total volume of 25 μL, containing 12.5 μL of the PCR Premix, 2.5 μL of the forward and reverse primers, 50 ng of the template DNA, and PCR-grade water to adjust the volume. Thermal cycling was as follows: initial denaturation at 98°C for 30 s; then 35 cycles of denaturation at 98°C for 10 s, annealing at 54°C for 30 s, and extension at 72°C for 45 s; followed by a final extension at 72°C for 10 min. The amplicons were analyzed by electrophoresis on 2% agarose gels and were purified by means of AMPure XT beads (Beckman Coulter Genomics, Danvers, MA, United States). The Quant-iT PicoGreen dsDNA Assay Kit was then employed. The library was quantified on a Qubit^®^2.0 fluorometer (Invitrogen, Carlsbad, CA, United States). The amplicon pools were prepared for sequencing, and size and quantity of the amplicon library were evaluated on an Agilent Bioanalyzer 2100 system with the Library Quantification Kit for Illumina (Kapa Biosciences, Woburn, MA, United States). The MiSeq instrument (Illumina, San Diego, CA, United States) was used to sequence 250-bp paired-end reads in two separate runs.

### Data Analysis

Paired-end reads were assigned to samples based on the unique barcodes, and the barcode and primer sequence were then removed. Paired-end reads were merged in the PEAR software (Paired-End reAd mergeR, Version 0.9.6). Raw reads were filtered to obtain high-quality clean reads under specific conditions of filtration via the FastQC (Version 0.10.1). Reads were scanned by the sliding window method, and the default value was 6 bp. In cases where the average phred quality score in the window was less than 20, sequences were truncated from the beginning of the window to the 3′ end. After truncation, reads shorter than 100 bp and with more than 5% ambiguous bases were excluded from the analysis. Moreover, the chimeric sequences were filtered by means of the VerSeach software (Version 2.3.4). Sequences were clustered into operational taxonomic units (OTUs; defined by 97% similarity) in VerSeach. Taxonomic data were assigned to the representative sequences (each OTU) using the RDP (Ribosomal Database Project) classifier. PyNAST software was used for multiple sequence alignment and to study the phylogenetic relationship between different OTUs. The α-Diversity that included two indices (Shannon and Chao1) was employed for the analysis of species diversity, and all indices were calculated in the QIIME software (Version 1.8.0). Excel 2007 (Microsoft Corp., United States) was used for graphics of the icewine yeast species abundance (mean values). The raw fungal ITS sequence data were deposited in the Sequence Read Archive (SRA) of the National Center for Biotechnology Information (NCBI) database under accession number PRJNA503950.

## Results

### Basic Chemical Analysis of the Composition of the Icewines

The spontaneous and inoculated fermentation processes were simultaneously terminated on day 30, and the basic chemical composition of the icewines was determined. The pH was 3.98, ethanol content was 9.8% (v/v) and residual sugar contents were 132.6 g/L in the icewine produced by spontaneous fermentation, whereas pH, ethanol, and sugar contents were 3.95, 12.6% (v/v), and 85.2 g/L, respectively, in the icewine produced by inoculated fermentation. The ethanol yields were clearly higher in the icewines produced by inoculated fermentation than those produced by spontaneous fermentation.

### Yeast Population Diversity and Dynamic Changes During the Fermentation Processes by the Culture-Dependent Method

A total of 338 strains were isolated at different stages of the spontaneous and inoculated fermentation processes and were differentiated based on colony color and morphology on WL nutrient agar (Cavazza et al., 1992); 21 different morphotypes were observed. The ITS region and the 26S rDNA D1/D2 domain of the representative strains were then sequenced for species identification. Twelve genera were detected, including *Aureobasidium*, *Candida*, *Cryptococcus*, *Filobasidium*, *Hanseniaspora*, *Pichia*, *Rhodotorula*, *Saccharomyces*, *Sporidiobolus*, *Torulaspora*, *Zygoascus*, and *Zygosaccharomyces*. The species found included *Aureobasidium pullulans*, *Candida californica*, *Candida railenensis*, *Candida salmanticensis*, *Candida smithsonii*, *Candida carpophila*, *Candida zemplinina*, *Cryptococcus flavescens*, *Cryptococcus magnus*, *Filobasidium floriforme*, *Hanseniaspora uvarum*, *Pichia kudriavzevii*, *Pichia occidentalis*, *Rhodotorula babjevae*, *Rhodotorula glutinis*, *Saccharomyces uvarum*, *Saccharomyces cerevisiae*, *Sporidiobolus pararoseus*, *Torulaspora delbrueckii*, *Zygoascus meyerae*, and *Zygosaccharomyces parabailii.* Table 1 provides an overview of the isolated representative strains, their ITS region and D1/D2 domain fragment sizes, and their GenBank accession numbers.

**Table 1:** ITS region and D1/D2 domain fragment sizes of the representative isolates and their GenBank accession numbers.

Isolate No.	Size (bp) (ITS region)	Accession No. (ITS region)	Size (bp) (D1/D2 domain)	Accession No. (D1/D2 domain)	Species
SSFB-3 10^−2^II3	549	MK131150	—	—	*Aureobasidium pullulans*
SSFB-2 10^−2^II7	406	MK131151	—	—	*Candida californica*
SSFC-1 10^−4^II5	582	MK131152	516	MK138601	*Candida railenensis*
SSFG-2 10^−4^II1	365	MK131153	—	—	*Candida salmanticensis*
SSFB-1 10^−2^II1	521	MK131154	—	—	*Candida smithsonii*
SSFD-3 10^−4^II5	510	MK131155	548	MK138602	*Candida carpophila*
SSFA-3 10^−3^II3	406	MK131156	—	—	*Candida zemplinina*
SZ-1 10^−2^II5	483	MK131157	553	MK138603	*Cryptococcus flavescens*
SZ-1 10^−2^I6	574	MK131158	—	—	*Cryptococcus magnus*
SSFB-2 10^−2^I1	598	MK131159	—	—	*Filobasidium floriforme*
SZ-2 10^−2^I2	—	—	481	MK138604	*Hanseniaspora uvarum*
SSFD-2 10^−5^II1	453	MK123420	549	MK138605	*Pichia kudriavzevii*
SSFG-1 10^−3^II2	392	MK123421	484	MK138606	*Pichia occidentalis*
SSFB-2 10^−2^II5	564	MK123422	—	—	*Rhodotorula babjevae*
SZ-2 10^−3^II3	554	MK123423	—	—	*Rhodotorula glutinis*
SSFE-3 10^−4^II4	—	—	521	MK138607	*Saccharomyces uvarum*
SSFE-2 10^−5^I1	790	MK123424	—	—	*Saccharomyces cerevisiae*
SZ-2 10^−2^I7	537	MK123425	—	—	*Sporidiobolus pararoseus*
SZ-2 10^−3^II2	743	MK123426	—	—	*Torulaspora delbrueckii*
SSFD-3 10^−5^II5	506	MK123427	—	—	*Zygoascus meyerae*
SZ-2 10^−3^I1	403	MK123428	—	—	*Zygosaccharomyces parabailii*

**FIGURE 1:**
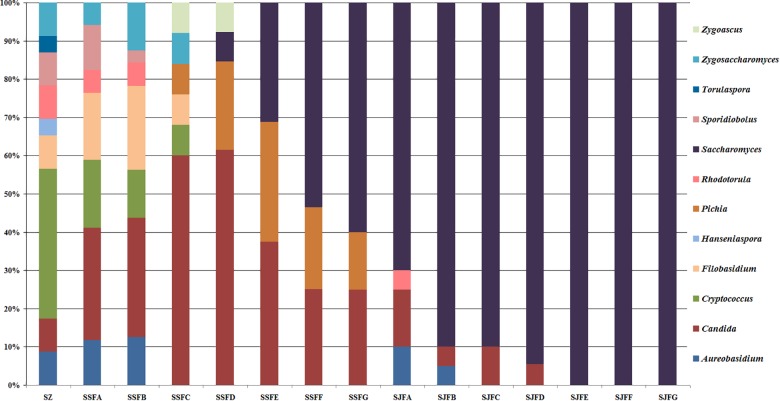
Histograms with the relative abundance of the yeasts isolated at each sampling point during spontaneous and inoculated fermentation processes (at the genus level).

Figure 1, 2 show changes in the isolated representative strains at genus and species levels during the spontaneous and inoculated fermentation processes. During the spontaneous fermentation, *Candida* was present during the whole process, and the relative abundance of the genus changed dramatically. The relative abundance gradually increased and then decreased, peaking at 61.6% on day 7 (SSFD), and accounted for 25.0% of the population on day 30 (SSFG). In the genus *Candida*, *C. zemplinina* was detectable at all sampling points (8.7–23.5%), representing 20.0% of the population at the end of the spontaneous fermentation (SSFG). *C. carpophila* and *C. californica* were detected on days 1–7 and days 2–21, respectively. The relative abundance of both species initially increased and then decreased, with their highest abundance levels being 28.0% (SSFC, day 4) and 18.8% (SSFE, day 14), respectively. *Pichia* (*P. kudriavzevii* and *P. occidentalis*) was detected from day 4 (SSFC; 8%) to day 30 (SSFG; 15%) and increased initially and then decreased, with the highest abundance being 31.3% (SSFE, day 14). *Cryptococcus* (*Cry. flavescens* and *Cry. magnus*) was the predominant genus at the beginning (SZ; 39.1%) and gradually dropped to 8.0% (SSFC, day 4), and eventually became undetectable. Similar to *Cryptococcus*, *Filobasidium* (*F. floriforme*) and *Zygosaccharomyces* (*Zygos. parabailii*) were found in the early fermentative stage (days 0–4), with their highest abundance levels being detected on day 2 (SSFB) and representing 21.9 and 12.5% of the population, respectively. *A. pullulans*, *Spo. pararoseus* and *Rhodotorula* (*R. babjevae* and *R. glutinis*) were all detectable at the first three sampling points (SZ, SSFA, and SSFB) and were absent later. Moreover, *H. uvarum* and *T. delbrueckii* were found only on day 0 (SZ) at low abundance (4.3%). *Saccharomyces* was first observed (7.7%) on day 7 (SSFD), and gradually increased in abundance to become the dominant genus in the later fermentative stages (days 21 and 30). The highest abundance was 60.0% (*S. cerevisiae* accounted for 45.0%) at the end of the spontaneous fermentation (SSFG).

**FIGURE 2:**
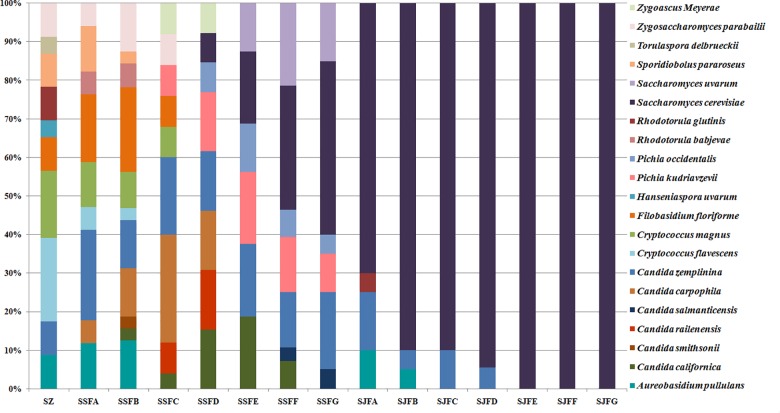
Histograms with the relative abundance of the yeasts isolated at each sampling point during spontaneous and inoculated fermentation processes (at the species level).

During the inoculated fermentation, *A. pullulans*, *C. zemplinina*, and *R. glutinis* were observed and their abundance levels decreased in the early stage. In the middle and later stages, other species were not detectable, with the exception of *S. cerevisiae.* From day 1 to 30, the abundance of *S. cerevisiae* gradually increased from 70.0% to 100.0%. Notably, *S. cerevisiae* was the predominant species during the whole process.

The yeast cell count in ice grape juice (SZ) was approximately 9 × 10^3^ CFU/mL. During the spontaneous and inoculated fermentation processes, the yeast cell count initially increased and then decreased. Table 2 presents the changes of yeast cell count and residual sugar content during the spontaneous and inoculated fermentation processes. In the spontaneous fermentation process, tiny bubbles were observed on the surface on day 4 (SSFC); while in the inoculated fermentation process, obvious bubbles appeared a day after inoculated commercial yeasts (SJFA).

**Table 2:** Changes of the yeast cell count and the residual sugar content during the spontaneous and inoculated fermentation processes.

	Day 1	Day 2	Day 4	Day 7	Day 14	Day 21	Day 30
SSF^a^	1.2 × 10^4^	2.5 × 10^4^	1.0 × 10^5^	2.8 × 10^6^	1.3 × 10^7^	8.5 × 10^6^	1.1 × 10^6^
SJF^a^	2.0 × 10^7^	4.5 × 10^7^	4.7 × 10^7^	3.0 × 10^7^	1.0 × 10^7^	4.0 × 10^6^	1.0 × 10^6^
SSF^b^	327.7 ± 1.2	319.7 ± 4.1	306.8 ± 1.4	282.2 ± 2.0	231.4 ± 1.2	172.4 ± 0.7	132.6 ± 1.9
SJF^b^	300.0 ± 1.6	260.6 ± 1.3	210.0 ± 2.6	168.9 ± 0.7	130.4 ± 0.6	99.4 ± 1.4	85.2 ± 1.1

*^a^Indicates the yeast cell count; values are expressed as CFU/mL. ^b^Indicates the residual sugar content; values are expressed as g/L.*

### HTS Data Analysis

HTS of the ITS2 region genes was conducted to analyze fungal community structure during the spontaneous and inoculated fermentation processes. Table 3 presents the summary of HTS data. The highest numbers of raw and clean reads were detected on day 4 of the spontaneous fermentation, and were 60,214 and 58,849 (SSFC), respectively. In contrast, the lowest numbers of raw and clean reads were 14,013 and 13,564 (SJFC), and these were observed on day 4 of the inoculated fermentation. The highest and lowest Shannon diversity indices were observed in SSFB (4.02) and SJFF (0.33) samples, respectively. The Chao 1 richness index was the highest and lowest for SSFE (71.78) and SJFD (17.25) samples, respectively.

**Table 3:** Summary of HTS data, including the number of raw and clean reads, Shannon diversity index and Chao1 richness index for fungal ITS rDNA libraries from samples at different stages of the spontaneous and inoculated fermentation processes.

	SZ	SSFA	SSFB	SSFC	SSFD	SSFE	SSFF	SSFG	SJFA	SJFB	SJFC	SJFD	SJFE	SJFF	SJFG
Raw reads	27189	38470	32279	60214	42241	34191	30353	36336	40829	34864	14013	40106	45427	46556	18623
Clean reads	26871	37830	31766	58849	41018	33535	29494	35485	39964	34030	13564	39083	44437	45461	18245
Shannon	3.30	3.80	4.02	2.23	1.68	3.36	2.69	2.34	0.42	0.82	1.01	0.47	0.42	0.33	0.56
Chao	52.83	61.00	65.20	65.20	64.98	71.78	65.98	60.03	44.17	40.01	28.87	17.25	20.86	20.15	32.11

*Shan, Shannon diversity index; Chao, Chao1 richness index.*

**FIGURE 3:**
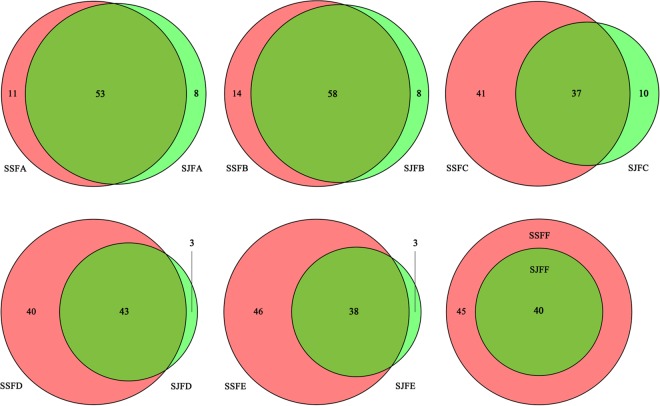
Venn diagrams of the number and relation of OTUs at each sampling point during the inoculated and spontaneous fermentation processes.

Figure 3 depicts a group of Venn diagrams highlighting the comparison of the OTUs at the same sampling point during the spontaneous and inoculated fermentation processes, and the OTUs obtained dynamically changed. On day 1, there were 64 OTUs in the spontaneous fermentation (SSFA) and 61 in the inoculated fermentation (SJFA), whereas 53 OTUs were identical between the two fermentation types. The number of OTUs present in SJFA but not in SSFA was 8, representing 11.1% of the total of OTUs in SSFA and SJFA. In SSFB and SJFB, there were 72 and 66 OTUs, respectively; the OTUs in the inoculated fermentation but not in the spontaneous fermentation accounted for 10% of all OTUs on day 2. From day 4 to day 14, the number of OTUs in the spontaneous fermentation increased from 78 (SSFC) to 84 (SSFE), while that in the inoculated fermentation decreased from 47 (SJFC) to 41 (SJFE); moreover, the percentage of the OTUs in the inoculated fermentation but not in the spontaneous fermentation gradually decreased from 11.4 to 3.4%. In the later fermentative stages (days 21–30), the number of OTUs in the spontaneous (SSFF and SSFG) and inoculated (SJFF and SJFG) fermentations was 45 and 40; it was notable that the number of OTUs in the inoculated fermentation but not in the spontaneous fermentation was 0. In summary, the number of OTUs increased initially and then decreased in the spontaneous fermentation, eventually reaching a peak at 84 on day 14. In the inoculated fermentation, the number of OTUs increased initially and then decreased gradually, but the highest value of OTUs was 66 on day 2. The percentage of the OTUs in the inoculated fermentation but not in the spontaneous fermentation decreased and was 0 from day 21 to the last sampling day (day 30).

**FIGURE 4:**
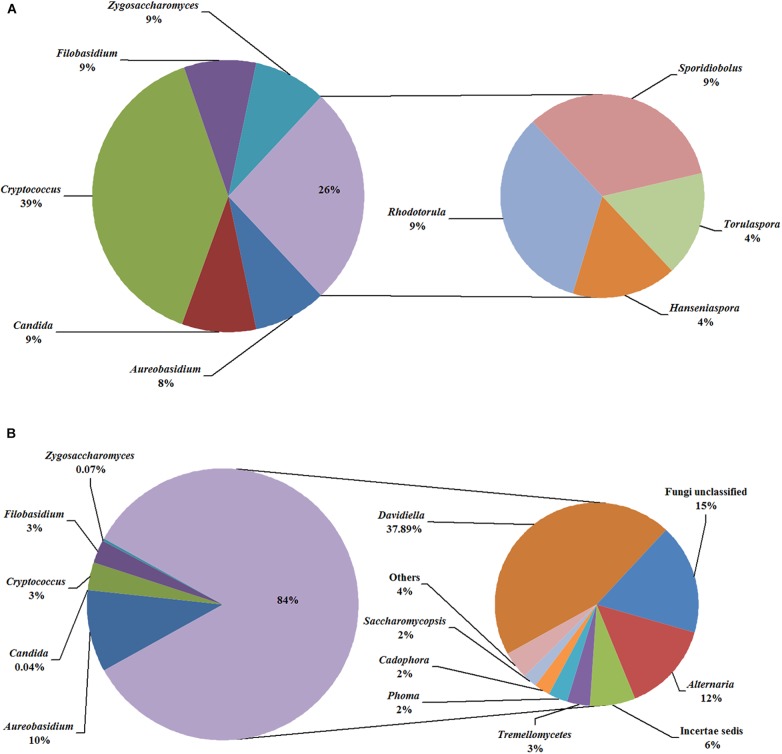
Relative abundance of the genera detected in the ice grape must (SZ) by culture-dependent **(A)** and HTS **(B)** methods. The “others” group includes *Hanseniaspora*, *Rhodotorula*, and so on.

In the fermentation processes, the presence of three phyla along with unidentified and unclassified ones was suggested; 36 genera along with unidentified and unclassified ones; 56 species with unidentified and unclassified ones. The HTS method revealed a more complex mycobiota than the culture-dependent method did in the fermentations. E.g., Figure 4 depicts the genera detected in ice grape juice (SZ) by the culture-dependent and HTS methods; except for *Aureobasidium*, *Candida*, *Cryptococcus*, *Filobasidium*, and *Zygosaccharomyces*, more genera were detected by the HTS method.

**FIGURE 5:**
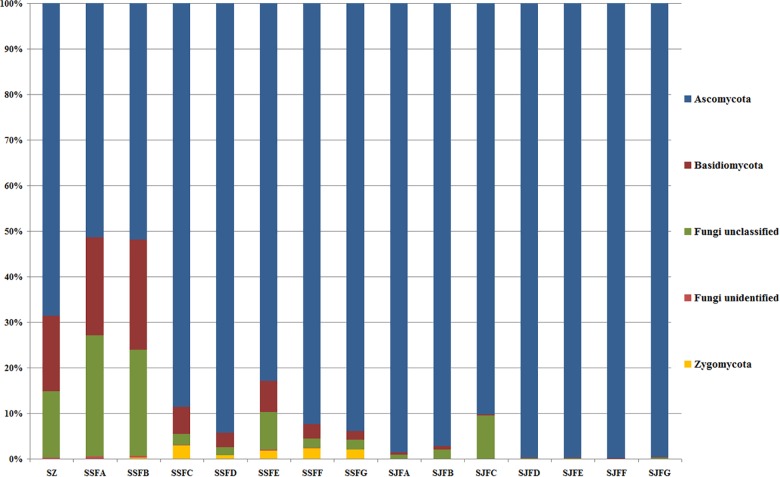
Histograms with the relative abundance of the yeasts during spontaneous and inoculated fermentation processes (at the phylum level) according to HTS analysis.

**FIGURE 6:**
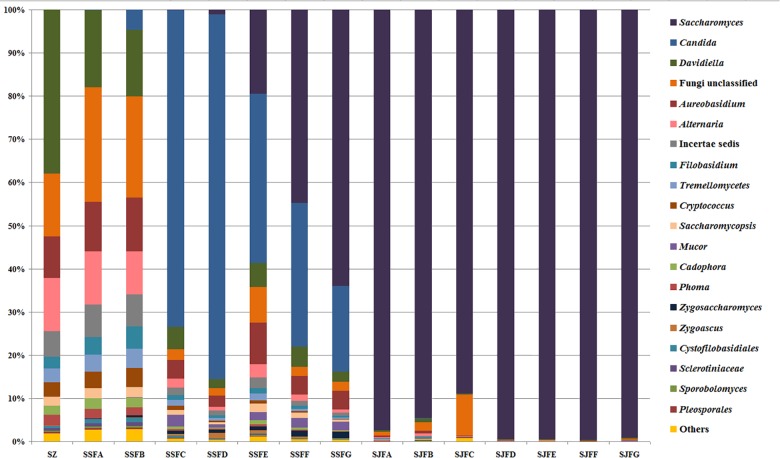
Histograms with the relative abundance of the yeasts during spontaneous and inoculated fermentation processes (at the genus level) according to HTS analysis.

**FIGURE 7:**
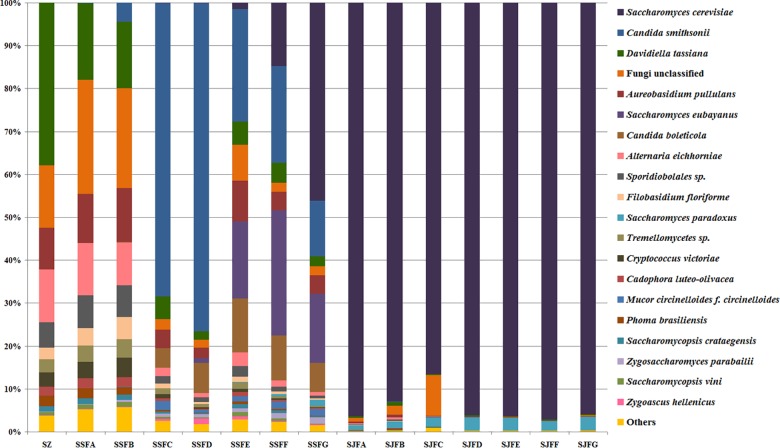
Histograms with the relative abundance of the yeasts during spontaneous and inoculated fermentation processes (at the species level) according to HTS analysis.

An obvious dynamic change in the yeast population was observed by the HTS method during the spontaneous and inoculated fermentation procedures. Figure 5–7 show fungal diversity and community changes at phylum, genus, and species levels (top 20) during the spontaneous and inoculated fermentation processes. A high abundance of Ascomycota was detected at all sampling points. This was the dominant phylum, as it reached the highest value of 94.30% on day 7 (SSFD) and the lowest value of 51.33% on day 1 (SSFA) in the spontaneous fermentation; it represented over 90% in all samples of the inoculated fermentation. The highest (24.22%) and lowest (1.89%) abundance levels of Basidiomycota were detected on days 2 (SSFB) and 30 (SSFG) of the spontaneous fermentation, respectively. Moreover, a low abundance of Zygomycota was detected (Figure 5).

*Candida* was observed at all sampling points, and its abundance levels were dramatically changed during the spontaneous fermentation. The relative abundance of *Candida* initially increased and then decreased, and became the dominant genus on days 4 and 7 (SSFC and SSFD), with the highest abundance being 84.44% on day 7 (SSFD). In the genus *Candida*, *C. smithsonii* showed significant changes, and gradually increased and then decreased during the spontaneous fermentation, with the highest abundance being 76.5% (day 7, SSFD). The abundance of *Candida boleticola* also increased initially, but then decreased, with the highest (12.6%) abundance detected on day 14 (SSFE). *A. pullulans* was also found at all sampling points and reached the highest abundance of 12.55% on day 7 (SSFD) and the lowest of 2.52% on day 2 (SSFB). *F. floriforme* and *Cryptococcus victoriae* were detected at all sampling points, with their highest abundance values being 4–6% on day 2 (SSFB) and the lowest observed on day 30 (SSFG, <1%). The abundance of *Saccharomyces* gradually increased and it became the dominant genus in the later fermentative stages, representing 63.95% of the population at the end of the spontaneous fermentation (SSFG). In the genus *Saccharomyces*, *S. cerevisiae*, *Saccharomyces eubayanus* and *Saccharomyces paradoxus* were observed during the spontaneous fermentation. *S. cerevisiae* abundance gradually increased in the middle and later periods and accounted for 46.19% of the population on day 30 (SSFG). *S. eubayanus* was detected on days 7–30 and its abundance initially increased and then decreased, with the highest abundance detected being 29.12% on day 21 (SSFF). *S. paradoxus* was present on days 14–30 at low abundance levels (<2%).

During the inoculated fermentation, *Saccharomyces* was the predominant genus during the whole process, and its abundance levels at all sampling points were above 90%, with the exception of SJFC (88.95%). *S. cerevisiae* and *S. paradoxus* were observed, and their highest abundance levels were 95.95% (SJFG) and 3.06% (SJFD), respectively. *A. pullulans* was also found at all sampling points, with the highest (9.73%) and lowest (0.1%) abundance levels detected at the beginning and end of the inoculated fermentation (SZ, SJFG), respectively.

### Comparison of Yeast Diversity and Dynamics Between During the Spontaneous and Inoculated Fermentation Processes

Clearly, yeast diversity in the spontaneous fermentation is much richer than that in the inoculated fermentation in both the culture-independent and HTS results. In detail, 12 genera (21 species) were detected by the culture-independent method in the spontaneous fermentation, and there were four genera (four species) in the inoculated fermentation.

During the spontaneous fermentation, non-*Saccharomyces* yeasts were predominant in the initial and middle stages, whereas *Saccharomyces* dominated in the later fermentative stages. However, *Saccharomyces* was the predominant genus during the whole inoculated fermentation process, and non-*Saccharomyces* yeasts were detectable in the early stages and then almost disappeared.

## Discussion

In this study, an obvious succession of yeast community was detected during the fermentation processes. To the best of our knowledge, this is the first study on the comparison of yeast diversity and the dynamics between during the spontaneous and inoculated fermentation of icewine using the cultivation approach (WL nutrient agar) and HTS approaches. During the spontaneous fermentation, non-*Saccharomyces* yeasts and the non-yeast fungi decreased in abundance, whereas *Saccharomyces* was gradually increased. This phenomenon can be explained as follows: the increase in ethanol concentration with the increase of fermentation time prevented the growth of non-*Saccharomyces* yeasts and non-yeast fungi (Fleet, 2006; Mills et al., 2008). The changes of non-*Saccharomyces* yeasts during the inoculated fermentation can be explained that the inoculated *S. cerevisiae* suppressed the growth of autochthonous non-*Saccharomyces* yeasts and performed rapid and complete fermentation (Urso et al., 2008).

In terms of non-*Saccharomyces* dynamics in the spontaneous and inoculated fermentation processes, it clearly decreased from the initial to the final stages. Of particular interest was the dynamic change of *Candida. C. zemplinina* was detected at all sampling points of the spontaneous fermentation by the culture-dependent method, which is in agreement with our previous study (Li et al., 2018). Notably, *C. zemplinina* was consistently detected in the early stage (days 0–7) of the inoculated fermentation. This is because *C. zemplinina* can grow at high sugar concentrations, at low temperatures and in the presence of ethanol (Sipiczki, 2003, 2004); and it can participate actively in fermentative production of sweet wine (Urso et al., 2008). Moreover, in previous studies, *C. zemplinina* was used to participate in mixed fermentation processes with *S. cerevisiae* that can reduce ethanol and acetic acid content and improve the aroma profile of wine (Englezos et al., 2017, 2018). The dynamic change of *C. californica* in the spontaneous fermentation was obvious according to the cultivation method. *C. californica* had been found in different wine-growing regions before (Vaudano et al., 2018; Aplin et al., 2019), and the latest studies have revealed that *C. californica*, when applied to inoculated fermentation with *S. cerevisiae*, can produce lower concentrations of residual sugar and ethanol as compared to fermentation with only *S. cerevisiae* (Aplin et al., 2019). Additionally, *C. carpophila* and *C. railenensis* were present in the early stages of the spontaneous fermentation; they have been detected in wine-growing regions before (Brežná et al., 2010; Brysch-Herzberg and Seidel, 2015; Drumonde-Neves et al., 2017). In further studies, the autochthonous strains of *Candida* can be screened on the basis of their outstanding enzymatic activities that affect and modulate the chemical and aromatic profile; these enzymatic activities are crucial to producing icewine by “multi-species” fermentation with unique characteristics.

Moreover, *Cry. victoriae* was detected at all sampling points in the spontaneous fermentation by HTS, but not by cultivation. Alessandria et al. (2013) also stated that *Cry. victoriae* was consistently detected during the spontaneous fermentation by the PCR-DGGE method (Alessandria et al., 2013). *Pichia* was detected in the spontaneous fermentation by cultivation, but not by HTS; it has been found in different wine-growing regions of the world (Brysch-Herzberg and Seidel, 2015; De Filippis et al., 2017; Bučková et al., 2018). Studies have revealed that *P. kudriavzevii* can positively influence the aroma and flavor of wine and has the potential to be used in winemaking (Del Monaco et al., 2014). *H. uvarum* and *T. delbrueckii* were often observed on grapes and in wine fermentation (Alessandria et al., 2013; De Filippis et al., 2017; Vaudano et al., 2018), but they were found only in the ice grape juice by the culture-dependent method in this study.

Interestingly, *A. pullulans* was detected both in the spontaneous and inoculated fermentation processes. It is a ubiquitous fungus in the environment (Singh et al., 2008), and is the yeast-like fungus and does not have the ability to spoil wine (Alessandria et al., 2013). In this study, it was detected in the initial stages of the spontaneous and inoculated fermentation processes but not observed in the later stages by the cultivation method. Nonetheless, it was detected throughout the entire fermentation process by HTS. Alessandria et al. (2013) stated that *A. pullulans* was found throughout the spontaneous fermentation process of icewine at the RNA level by the PCR-DGGE method; however, *A. pullulans* was not detected in the inoculated fermentation process; in fact, the starter profiles were only showed in the inoculated fermentation process by PCR-DGGE method (Alessandria et al., 2013). It has been reported that PCR-DGGE is not useful for detecting species with densities below 10^3^ CFU/mL (Portillo and Mas, 2016). Therefore, the HTS method is more comprehensive than the PCR-DGGE method for analyzing the microbial diversity of fermented foods.

Thus far, ITS-rDNA as a target amplicon was generally selected for amplification in the studies on fungal populations during wine fermentations (Bokulich et al., 2014; Pinto et al., 2015). In our previous research, the ITS1 region of the samples was selected for amplification; the notably “incertae sedis” result was obtained after HTS, and some species were detected by cultivation method but not by HTS (Li et al., 2018). In addition, the ITS2 region was suggested to be more variable than ITS1 (Bazzicalupo et al., 2013; Sommermann et al., 2018). Because of the above issues, the ITS2 region of the samples was selected for amplification in this study; however, “Fungi unclassified” and “Incertae sedis” were also observed in HTS results, and some species were found by cultivation method but not in HTS results. Regarding “Fungi unclassified” and “Incertae sedis,” that is because the databases used for fungal comparisons generally have limited coverage and need extended follow-up in the future (Ercolini, 2013).

Regarding the differences in the results between the cultivation and HTS methods, Mendoza et al. (2017) also stated that HTS results revealed a large number of yeast species not recovered by culture-dependent method; moreover, the cultivation method detected very few yeast taxa, most of them corresponding with very few or zero reads in HTS analysis (Mendoza et al., 2017). The differences in the results by the two methods have also been reported in previous studies of foods and wines (David et al., 2014; Greppi et al., 2015). In this study, it can be explained by the limitations of the two methods; the cells in a sublethal or injured physiological state can be absent in the results of the cultivation (Rantsiou et al., 2005; Mendoza et al., 2017); the bias of different primers exist, the sequencing depth of the HTS method may be insufficient, and preferential amplification of shorter fragments may occur during PCR, and so forth (Ercolini, 2013; De Filippis et al., 2017; Mendoza et al., 2017; Karst et al., 2018). Because of the above issues, both the culture-dependent and HTS methods were employed to analyze yeast diversity and dynamics during the spontaneous and inoculated fermentation processes, which is a more comprehensive strategy.

In addition to wine yeasts, we found some other fungi in the fermentation by the HTS method, mainly *Davidiella* and *Alternaria*. They were also found in grape must or fermented wine samples of different wine-making regions (Bokulich et al., 2014; Pinto et al., 2015). *Davidiella tassiana* (other name is *Cladosporium herbarum*) is a fungal plant pathogen, which causes a common disease of grapevines (*Cladosporium* rot) (Latorre et al., 2011). Previous research indicates that this pathogen can reduce the yield and affect the quality of wines (Briceño et al., 2009). It is found particularly in the vines that are harvested very late (Briceño and Latorre, 2008). In the present study, the ice grapes used for making icewine were harvested in December, which was later than the harvest time of common wine grapes. *Alternaria* has been the main component of the wine grape mycobiota in several wine-making regions worldwide (Prendes et al., 2015; Bučková et al., 2018). *Alternaria* includes numerous species, and some of these species produce mycotoxins that can pose a potential health hazard for consumers. *Alternaria* toxins in wine, beer, and other foods have been reported (Fan et al., 2016). *Alternaria alternate* is the most prevalent species, which is the most important mycotoxin-producing fungus (European Food Safety Authority [EFSA], Arcella et al., 2016). Nevertheless, *Alternaria alternate* was not detected in our study, but *Alternaria eichhorniae* was found. Very few reports have studied *Alternaria eichhorniae*. Shabana et al. (1995) stated that *Alternaria eichhorniae* is a safe and effective bioherbicide for waterhyacinth (Shabana et al., 1995). In the present study, the highest abundance levels of *Davidiella* and *Alternaria* were both detected in raw ice grape juice. Thereafter, their abundance decreased clearly, and their abundance levels at the end of the spontaneous fermentation were low. The abundance decrease may be attributed to the inappropriate cultivation conditions for these species with the increase in fermentative time. Bokulich et al. (2014) stated that fungal profiles were dominated by filamentous fungi in the grape juice samples (close to 70%), and mentioned that many microorganisms inhabiting the grape surface cannot survive the low-pH, ethanolic, anaerobic conditions of wine fermentation processes, e.g., phytopathogenic fungi, but their metabolic activity can have long-ranging consequences (Bokulich et al., 2014). The abundance levels of *Davidiella* and *Alternaria* in the inoculated fermentation were all no more than 1% from day 1 to day 30. Comparison of *Davidiella* and *Alternaria* abundances during the spontaneous and inoculated fermentation processes shows that we have reason to believe that the inoculated fermentation icewine should be safer than the spontaneous fermentation icewine. To the best of our knowledge, the presence of *Davidiella* and *Alternaria* and their dynamic changes during icewine fermentation were revealed for the first time. The metabolites and mycotoxins in icewine should be researched in more detail in future works.

In this study, yeast diversity and dynamics during the spontaneous fermentation were found to be different from those in our previous research (Li et al., 2018). For example, *C. californica* and *C. carpophila* have been found in this study, but not in the previous research; *Metschnikowia* had been isolated from the samples of the previous research, but not in this study. Although the same grape variety was employed and the vineyards were both in northeast China in these two studies, different locations, years of sampling, and compositions of samples could explain these discrepancies. In the previous studies, different wine-growing regions were reported to maintain different microbial communities (called “microbial terroir”) (Bokulich et al., 2014; Gilbert et al., 2014).

## Conclusion

The combination of a microbial cultivation method and HTS approaches is useful for estimating across-the-board microbial diversity and dynamics during the spontaneous and inoculated fermentation processes of Vidal blanc icewine. Further studies on the oenological characteristics of autochthonous yeast strains and their contribution to the sensory quality, as well as the microbial safety of the dominant yeast strains found in this study, could help us to use them in co-inoculation trials to produce icewines with territorial characteristics and particular flavors.

## Data Availability

The datasets generated for this study are available in the Genbank and Sequence Read Archive (SRA) of the National Center for Biotechnology Information (NCBI) database, PRJNA503950.

## Author Contributions

JL and W-ZH designed the experiments. JL conducted the experiments and analyzed the experimental data. JL, W-ZH, and Y-PX wrote the manuscript.

## Conflict of Interest Statement

The authors declare that the research was conducted in the absence of any commercial or financial relationships that could be construed as a potential conflict of interest.

## Footnotes

1 http://www.ncbi.nlm.nih.gov

## Abbreviations

HTS: high-throughput sequencing
ITS region: internal transcribed spacer region
PCR-DGGE: polymerase chain reaction with denaturing gradient gel electrophoresis
rDNA: ribosomal DNA
WL nutrient agar: Wallerstein laboratory nutrient agar.
